# Spectral Emissivity and Thermal Conductivity Properties of Black Aluminum Films

**DOI:** 10.1021/acs.langmuir.4c03838

**Published:** 2025-01-02

**Authors:** Joris More-Chevalier, Jiří Martan, Taavi Repän, Sylvain Duprey, Petr Hruška, Michal Novotný, Petra Honnerová, Jan Kejzlar, Christophe Labbé, Morgane Poupon, Dejan Prokop, Daniil Nikitin, Xavier Portier, Přemysl Fitl, Julien Cardin, Raivo Jaaniso, Ján Lančok

**Affiliations:** † Institute of Physics, Czech Academy of Sciences, Na Slovance 2, 182 21 Prague 8, Czech Republic; ‡ Department of Physics and Measurements, University of Chemistry and Technology Prague, Technická 5, 166 28 Prague 6, Czech Republic; § New Technologies Research Centre (NTC), University of West Bohemia, 301 00 Pilsen, Czech Republic; ∥ Institute of Physics, University of Tartu, W. Ostwald St 1, Tartu 50411, Estonia; ⊥ CIMAP Normandie Université, ENSICAEN, UNICAEN, CEA, UMR CNRS 6252, 6 Boulevard Maréchal Juin, 14050 Caen Cedex 4, France; # Charles University, Faculty of Mathematics and Physics, V Holešovičkách 2, 180 00 Prague, Czech Republic

## Abstract

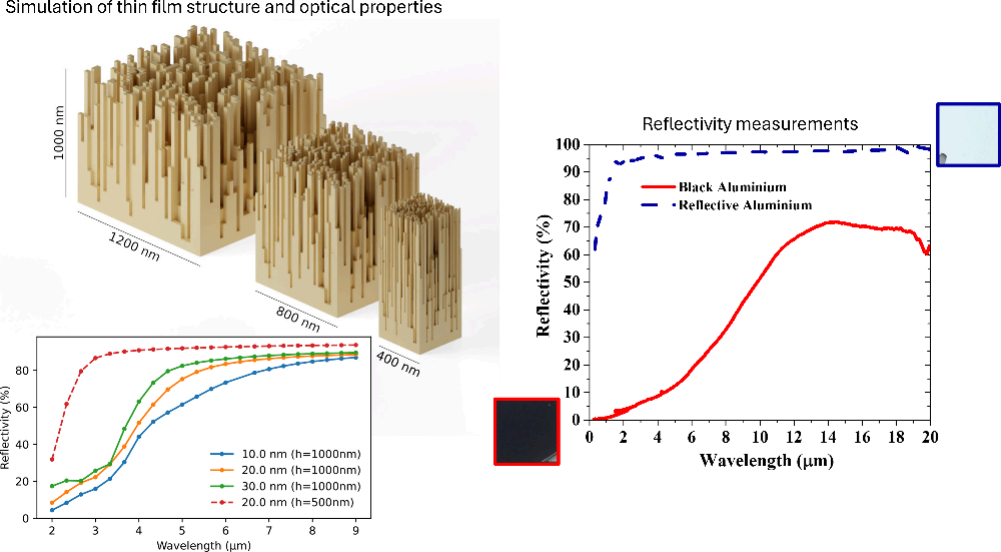

Black aluminum is a material characterized by high surface porosity due to columnar growth and exhibits unique optical properties that make it attractive for applications such as light trapping, infrared detection, and passive thermal radiation cooling. In this study, we correlate the structural and optical properties of black aluminum by comparing it with conventional reflective aluminum layers. These layers of varying thicknesses were deposited on fused silica substrates, and their optical properties were analyzed. COMSOL simulations, supported by experimental data, reveal that black aluminum’s structure leads to a significant reduction in visible light reflectivity and an increase in emissivity in the near- and mid-infrared ranges. This enhanced emissivity is partly due to the presence of aluminum nitride (AlN) grain boundaries and an oxidized surface layer. Optically, black aluminum differs significantly from reflective aluminum by presenting a reflectivity below 5% in visible wavelength and an average emissivity of approximately 0.4–0.5 from 1.2 to 20 μm. Thermally, it possesses approximately ten times lower thermal conductivity and doubles the volumetric heat capacity. These differences are attributed to its porous structure, nanoscale crystallites, and the presence of aluminum nitrides and oxides within the material.

## Introduction

The growing focus on renewable energy has reignited interest in developing efficient technologies for energy conversion. Of particular importance is the conversion of waste heat into usable electricity, which can be achieved through two main effects: thermoelectric and pyroelectric effects. Thermoelectric devices generate electricity under a steady temperature gradient via the Seebeck effect.^1−4^ In contrast, pyroelectric devices require temperature oscillations to operate.^5,6^

Among renewable energy sources, light can be used and converted to heat from a black coating to produce electrical energy. Several black metal coatings have been proposed and tested to provide rapid heat exchange from light sources for pyroelectric conversion and improve the pyroelectric detectors’ sensitivity.^7−9^ A twice higher value of pyroelectric current was measured from a pyroelectric coated with black gold and heated with near-infrared radiant heating.^7^ An improvement of 3.4 times in the measured pyroelectric electric voltage was observed using a graphene layer compared to a classical aluminum top electrode layer.^10^ Recently, a doubled variation of the polarization was observed when a light was applied to the surface of the stack pyroelectric structure based on Pb(Zr_*x*_Ti_1–*x*_)O_3_ with stable black aluminum on the top.^9^ Black coatings also improve the thermoelectric properties. For example, light conversion was reported with black coatings using black-Si as absorbers and plasmonic absorbers.^11−13^ In addition, a high light-trapping performance from surface nanostructured black-Si was prepared using the reactive ion etching method and then put on a thermoelectric module.^12^ Moreover, a stabilized temperature of 53.8 °C was achieved by applying a solar irradiance of 100 mW/cm^2^ on the black Si, which permitted the production of a voltage value of 60 mV across the thermoelectric module. We can notice also that plasmonic nanostructures for high-efficiency light absorbers (∼96.8%) were developed based on Al/AlN for solar energy conversion.^13^ Moreover, a temperature of ∼120 °C was reached, allowing us to get a voltage of 140 mV across a commercial thermoelectric module coated with the plasmonic nanostructures. Other black metals (BMs) have a very high potential for the topic of energy harvesting.^9,14−17^ Silver nanoplate aggregations exhibited a large absorbance of nearly 100% in the range from 400 to 1100 nm, which can be useful in various applications like solar energy harvesting, photothermic utility to wide-wavelength range antireflection, and cloaking.^14^ The high-trapping light structures are due to the introduction of porosity during the metal film growth from the presence of impurities.^18,19^ Several metals were investigated to obtain black or colored like gold, platinum, tungsten, copper, titanium, palladium, or aluminum.^20−27^ Several techniques have been employed to prepare BM layers like magnetron sputtering,^9,28−31^ thermal evaporation,^32−35^ electrodeposition,^36,37^ metal film deposition on dielectric substrates with imprinted antireflective black moth-eye-like nanostructures,^38,39^ and laser surface modifications.^15,27^ The generated high surface roughness structure from these BM films can be used to enhance the effective surface of a quartz crystal microbalance or chemiresistor for gas sensor applications.^35,40^

Black aluminum (B-Al) offers several advantages including excellent wear resistance, strong adhesion, and the ability to maintain its optical properties at temperatures above 300 °C.^29,30,41,42^ Studies using thermally stimulated desorption and exoelectron emission under ultrahigh vacuum conditions (*p* < 2 × 10^–7^ Pa) have revealed a link between sorption and desorption processes at the B-Al surface layer.^43^ Thermally stimulated desorption indicates two stages for the release of weakly bound atoms such as oxygen, nitrogen, and AlOH radicals. The first stage, below room temperature (RT), involves physical desorption. The second stage, above RT, involves the desorption of chemisorbed particles and the release of radicals, such as AlOH and AlN, at temperatures exceeding 200 °C. Recent studies show that mechanically intact B-Al films remain thermally stable up to at least 400 °C.^30^ However, spiral-shaped aluminum nanowires have been observed to grow on damaged or scratched B-Al surfaces after heating to 200 °C. While B-Al films possess valuable properties,^29−31,44^ there remains a lack of data on their thermophysical properties as well as on black metals (BMs) in general. Understanding these properties and their relationship to other factors is crucial, especially for applications in energy harvesting and thermal radiation cooling.

In this work, we present a comparative analysis of reflective (R-Al) and black aluminum (B-Al) films focusing on their optical properties, thermal conductivity, and emissivity. The relationship between their microstructures and their properties is also explored through different film thicknesses.

## Experimental and Methodology

### Film Depositions

Reflective (R-Al) and black aluminum (B-Al) films were deposited by pulsed DC magnetron sputtering on fused silica (FS) substrates. Substrates were fixed on an unheated substrate holder placed 100 mm from the target. A round-shaped magnetron aluminum target (Al purity of 99.99%) with a diameter of 100 mm was used for sputtering. A DC power supply (Huttinger 3000) combined with a pulse generator (MELEC) was used at an average power of 400 W, with a repetition frequency (f) of 10 kHz and a duty cycle of 50%. The deposition rate was equivalent for each film at 80 nm/min. The vacuum chamber was pumped down by a diffusion pump to a base pressure of ∼4 × 10^–3^ Pa. The magnetron discharge was kept in a reactive atmosphere of N_2_/Ar with ∼6% N_2_ for the B-Al films and in a pure Ar atmosphere for the R-Al films. The deposition pressure was kept constant at 0.5 Pa, regulated using a throttle valve at the high-vacuum pump gate valve. DC voltages were 415 and 365 V for R-Al and B-Al depositions, respectively. The thicknesses of the films were ∼330, ∼690, and ∼1090 nm for R-Al layers and ∼225, ∼540, and ∼950 nm for B-Al layers. Thicknesses were measured by contact profilometry.

### X-ray Diffraction

The XRD diffraction patterns were measured using the Empyrean Panalytical Series 2 diffractometer with a Cu K_α_ (λ = 1.5418 Å, 45 kV, 30 mA) X-ray tube and a PIXcel3D–Medipix3 area detector in open-slit mode and grazing incidence geometry (with an angle of incidence of 3°). A parallel beam was created using a Göbel mirror (graded W/Si crystal) placed in front of the primary beam. A horizontal mask of 8 mm, Soller slits of 0.04 rad and a fixed slit of 1/2° were used to ensure that the spot size is smaller than the sample width (<10 mm). A parallel plate collimator of 0.18° was placed in the path of the diffracted beam. The step size of 0.1° was chosen with a time step of 4 s. Jana2020 software was used to fit the XRD profile.^45^

### Scanning Electron Microscopy

SEM (Jeol, JSM 7200F) was run in SE mode with a 15 kV acceleration voltage to capture surface morphology images at RT. The ImageJ software was used to increase the surface morphology sizes of the grains/crystallites. The morphology sizes were selected manually by surrounding the considered area of the grains. A log-normal distribution was performed on each sample surface by extracting between 240 to 300 different grain sizes on three different images from the same sample at the same focus (6 μm × 5 μm image size).

### Atomic Force Microscopy

Measurements were carried out under ambient conditions using an atomic force microscope (NT-MDT, NTEGRA Prima). The AFM images had a resolution of 512 pixels × 512 pixels and were taken in amplitude modulation intermittent contact mode by a Multi75Al-G silicon AFM probe (BudgetSensors, *f*_0_ = 75 kHz, *k* = 3 N/m, nominal tip radius of 10 nm). The data were treated with the Gwyddion software.^46^

### Reflectivity and Emissivity Measurements

The optical properties of the films were evaluated by using a PerkinElmer Lambda 1050 spectrophotometer covering the UV–visible–NIR spectrum. Specular reflectance was measured using universal reflectance accessory (URA) mode. Prior to measurements, the system’s 100 and 0% baselines were calibrated. Measurements were taken at an incidence angle of 8°, with unpolarized light, using a beam size of 2 × 2 mm^2^ and a scanning rate of 1 nm per second.

A Specord 210 Plus dispersive spectrophotometer supplemented with an integrating sphere and an incidence angle of 8° from normal to the measured surface was used for reflectivity analysis in the visible and near-infrared spectral range. A Nicolet iS50 FTIR spectrometer equipped with an integrating sphere with an incidence angle of 12° from normal to the measured surface is used for reflectivity analysis in the spectral range of 1.5 to 20 mm.^47^ In both cases, the radiation reflected from a standard reference material with a known spectral reflectivity value and from the sample surface is compared, and the reflectivity of the measured sample is evaluated. The spectral emissivity is evaluated according to Kirchhoff’s law.^48^

### Thermal Conductivity Measurements

Thermal effusivity in various depths under the sample surface was determined by a measurement system based on pulsed photothermal radiometry described in detail in refs (49) and (50). It uses a pulsed laser to heat the material surface and a fast infrared detector to observe the temperature decrease after the laser pulse impact. From the temperature decrease with time in the nanosecond time range, the thermal effusivity at a depth of hundreds of nanometers was evaluated. Thermal effusivity is the square root of thermal conductivity, specific heat, and density, and it expresses the ability of the material to extract the heat from the surface to the material. The experimentally measured effusivity evolutions in time were analyzed and fitted to get the thermal conductivity and volumetric heat capacity.

### Simulation Methodology

The finite-element package COMSOL Multiphysics was used to perform full-wave simulations of porous structures. In the simulation, we used periodic boundary conditions in two directions. A custom Python code generated the simulation geometry with varying cell size and porosity (amount of air vs metal). For the results presented here, we used a regular 40 × 40 × 10 grid as the basis of the geometry, although we could also generate irregular geometries. By scaling the geometry in the *x*–*y* direction, we could vary the cell size (i.e., surface morphology size). The sample is illuminated with a normally incident plane wave. In the calculations here, the substrate has been neglected; i.e., the Al sample is surrounded by air on both sides.

## Results and Discussion

### X-ray Diffraction Patterns

**Figure 1:**
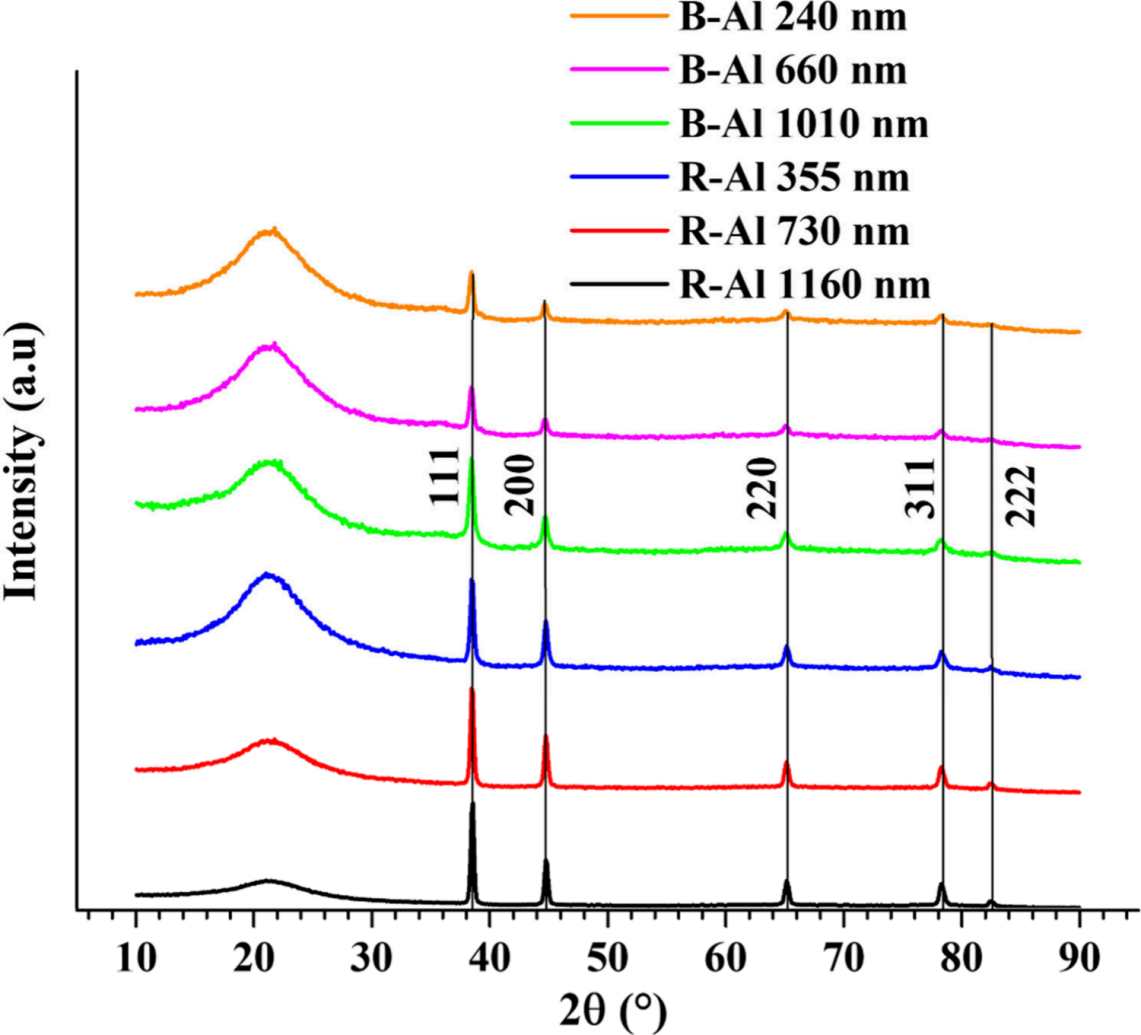
XRD diffraction patterns from R-Al and B-Al layers deposited on fused silica (FS) substrates as a function of their thicknesses. The solid lines indicate the Al peaks from a structure with the *Fm*3̅*m* space group and a lattice parameter *a* = 4.0490 Å.

The XRD θ–2θ scans over the 2θ range from 10 to 90° for both reflective aluminum (R-Al) and black aluminum (B-Al) layers, as a function of their thickness, are shown in Figure 1. The broad peak observed in all XRD patterns between 15 and 25° corresponds to the diffuse scattering from the amorphous silica substrate.^51,52^ For all films, the peaks align with the diffraction pattern of the aluminum (Al), which belongs to the *Fm*3̅*m* space group.^53^ The precise peak positions are listed in Table S1 and in the Supporting Information. Additionally, a weak extra peak can be observed at 2θ = 36.03° and 2θ = 35.94° for the 660 and 1010 nm B-Al layers, respectively, corresponding to the 002 orientation of the AlN phase presenting a Wurtzite-type structure with a *P*6_3_*mc* space group.^54^

The fits of R-Al 730 and 1160 nm show the expected results for relaxed Al films with lattice parameters of *a* = 4.0493 Å and *a* = 4.0497 Å, respectively, which are close to the bulk Al value of 4.0490 Å.^53^ On the other hand, the R-Al 355 nm layer exhibits a compressive stress with a fit lattice parameter of *a* = 4.0471 Å. Numerous studies have reported that residual stresses in metallic films are observed depending on the deposited film thickness and the deposition conditions.^55−58^ For Al thin films, stressed films have been observed at up to 300 nm when the film is deposited with a working pressure of 2 Pa.^57^ The compressive stress in sputtered Al thin films is usually attributed to the atomic pinning effect of the energetic particles hitting the films and producing defects and distortions in the lattice during the film growth.^57,59^

The B-Al layers exhibit an intrinsic stress that varies with thickness. Initially, a slight tensile stress is observed in the thinnest layer (240 nm), while both the 660 and 1010 nm layers show slight compressive stress. The tensile stress in the B-Al 240 nm layer might be due to the film’s tendency to become continuous.^57^ At this thin thickness, the introduction of N_2_ impurities during deposition may begin to affect the typical Al film growth at room temperature, which could explain results similar to those reported for equivalent Al films in the literature.^57,60,61^ In contrast, the B-Al 660 and 1010 nm show slight compressive stress in their lattice parameters of *a* = 4.0483 Å and *a* = 4.0477 Å, respectively. These findings differ from our previous results, where no lattice stress was reported for a B-Al film of 500 nm and tensile stresses were observed for films of 1.6 and 7.58 μm.^18,19^ The reasons for these discrepancies are not clear. They may come from the difference in the base pressures before deposition, which varied between experiments ranging from ∼2 × 10^–3^ Pa to 5 × 10^–3^ Pa. The base pressure influences the oxygen impurity concentration which can affect the induced stress in the deposited metallic layers as Al.^56−58,60−63^ However, this induced stress does not appear to significantly impact the outcome, which remains a black metal coating in all reported cases, including this study.

### SEM and AFM Images

**Figure 2:**
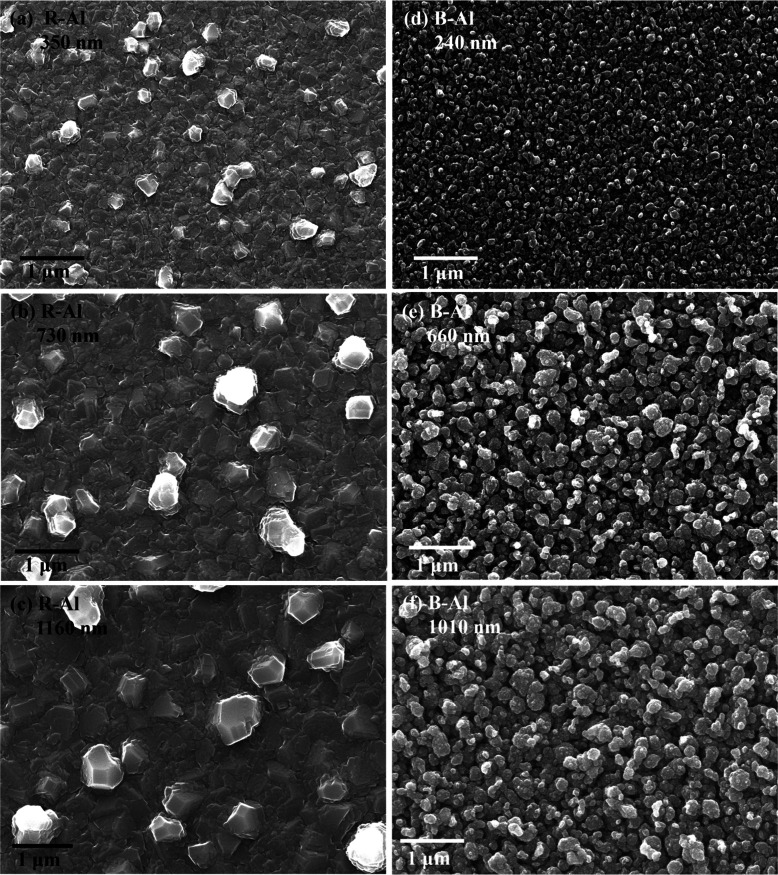
SEM images of the surface of R-Al films with a thickness of 355 nm (a), 730 nm (b), and 1160 nm (c). B-Al films with a thickness of 240 nm (d), 660 nm (e), and 1010 nm (f).

Figure 2 presents the SEM images of the surfaces of R-Al and B-Al films as a function of the film thickness. The surface morphologies of R-Al and B-Al films are consistent with those reported in our previous works.^19,29,30^ The main change is an increase in the size of the observed grain morphologies as a function of film thickness. This increase is coherent with the coalescence phenomena and with the competition between grains in the film growth, which tends to merge and grow crystallites/grains with the deposition time for a metal film deposited at room temperature.^18,63,64^ It has been demonstrated on several metals like Cu, V, and Al that the grain/crystallite size is correlated to the film thickness by a power law.^63^ For B-Al films, a noticeable change in grain morphology is observed between the 240 and 660 nm films, with larger grains at greater thickness. However, the change in the grain morphology seems less pronounced between the B-Al film at 660 nm and the B-Al film at 1010 nm.

**Figure 3:**
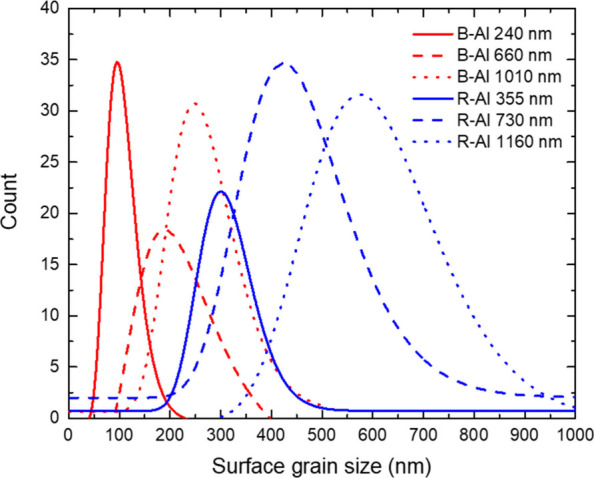
Surface distribution of grain size morphologies extracted from the SEM images of the R-Al and B-Al films.

Figure 3 shows the log-normal distribution of the grain size morphology extracted from the SEM images. The mean grain sizes are 310 ± 2, 449 ± 6, and 603 ± 12 nm for R-Al films of 355, 730, and 1160 nm. For B-Al films, the mean morphology grain sizes are 104 ± 4, 223 ± 7, and 264 ± 9 nm for thicknesses of 240, 660, and 1010 nm, respectively. A linear regression analysis of the mean grain size for R-Al films seems to suggest a linear relationship with thickness, with a standard error of the slope estimated to be around 4.5% (Figure S1). However, the relationship between grain size and thickness for B-Al films appears to be nonlinear, as indicated by a much higher slope error of approximately 17%. B-Al films consistently exhibit grain sizes that are 2 to 2.5 times smaller than those of R-Al films. This discrepancy is consistent with the known influence of nitrogen impurities on metal film growth, which tends to reduce atom mobility on the film surface, thereby affecting the film microstructure and reducing the grain size.^63,64^ R-Al films exhibit larger grain morphologies, which correlate with the improved XRD patterns observed in the previous section. This confirms that R-Al layers are denser and composed of larger crystallites, resulting in the formation of large grains on the film surface due to the Volmer–Weber growth mechanism.^18^

**Figure 4:**
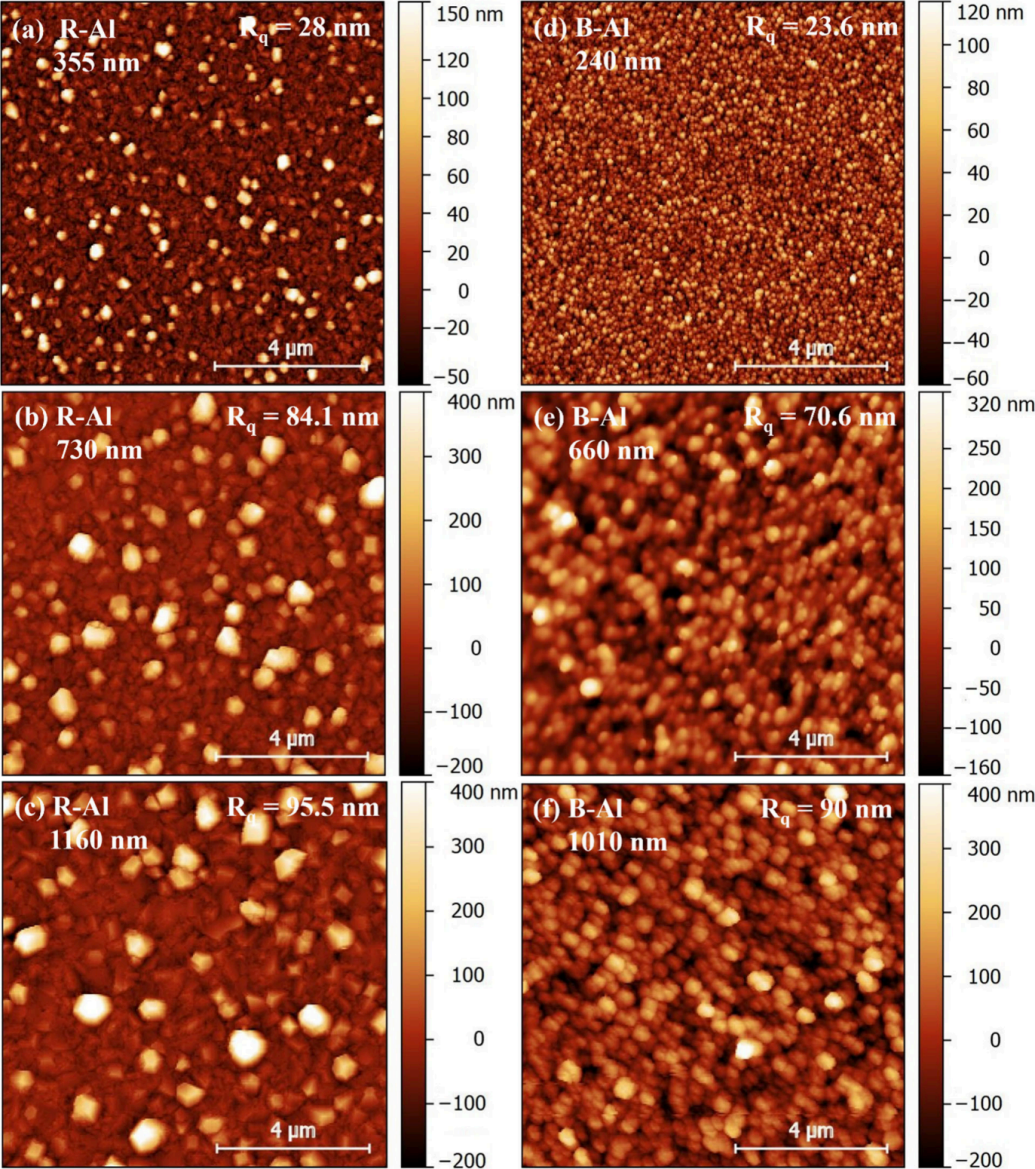
AFM images of the R-Al film surfaces in (a), (b), and (c) and B-Al film surfaces in (d), (e), and (f). The RMS surface roughness values *R*_q_ are indicated for each image.

Figure 4 presents AFM images of R-Al and B-Al film surfaces with corresponding RMS roughness (*R*_q_) values. The observed surface roughness deviates from typical findings but aligns with results from previous studies.^18,19,25,26^ This discrepancy may be explained by the thicker R-Al films compared with B-Al films deposited for the same duration. R-Al films exhibit a surface characterized by larger grains and slight roughness between them, while B-Al films show significant height variation, indicative of high porosity.

This difference tends to cause an increase in grain size for Al films with greater thicknesses.^63^ On the other hand, the significant variation in grain sizes observed and reported for R-Al films in Figure 2, Figure 3, and Figure 4 suggests that, in our work, the Al films follow a growth corresponding to films deposited close to zone T and not to films deposited in zone II, as described in the work reported by Petrov et al.^64^ Growth in zone T is associated with a rougher surface due to the greater diversity in grain sizes compared to films grown in zone II. This difference in the common Al film growth is explained, in this work, by the base pressure of ∼4 × 10^–3^ Pa in our deposition system, which increases the impurity concentration in the chamber and slightly modifies the film growth process.^43,44^ It has been reported that impurities during Al film growth lead to smaller grain sizes due to increased nucleation density on the substrate and reduced atom mobility.^63,64^ While the base pressure of ∼4 × 10^–3^ Pa is slightly higher than the base pressure usually reported in our previous work of ∼2 × 10^–3^ Pa,^18,19,29,35^ we believe that this minor difference does not significantly impact the overall conclusions of the reported work.

B-Al film surfaces in Figure 4(d), (e), and (f) show an increase in RMS film roughness values from *R*_q_ = 23.6 nm for a B-Al film of 240 nm to *R*_q_ = 90 nm for a B-Al film of 1010 nm. The RMS value of the B-Al 1010 nm film is close to the one reported previously of *R*_q_ = 100 nm measured on a B-Al film of 1630 nm.^19^ Overall, the surface morphologies and roughnesses of B-Al films in this study are consistent with those reported for similar B-Al films.^18,19,35^

### Optical Properties in the Visible Range

**Figure 5:**
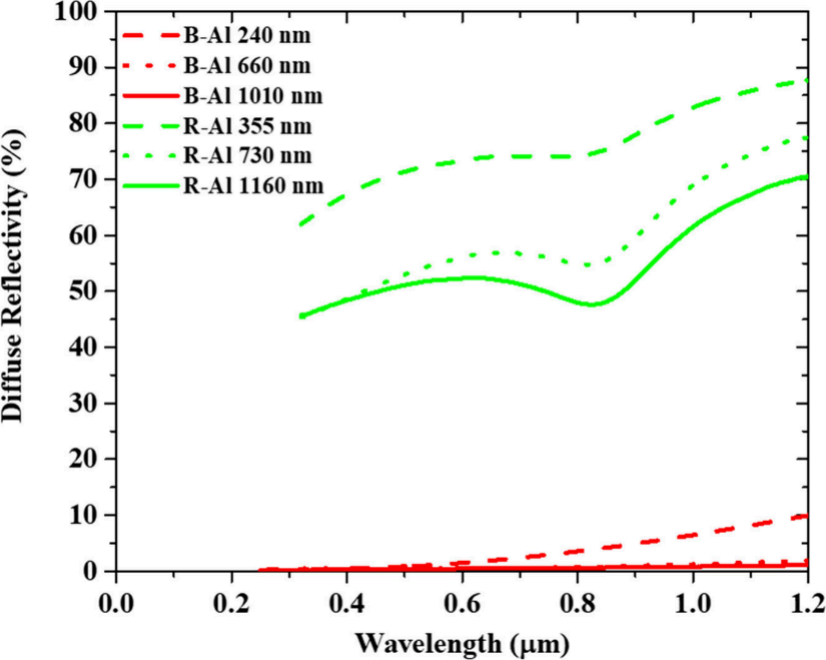
Diffuse reflectivity of R-Al films and B-Al films for each studied thickness.

Figure 5 presents the diffuse reflectivities of the R-Al films and B-Al films for the various thicknesses. A decrease in the reflectance intensity is observed with increasing R-Al thickness. The observed decrease in reflectance with increasing film thickness aligns with the known trend of increased surface roughness. This roughness enhances light absorption, as documented in previous studies.^65^ An absorbance peak is observed at ∼830 nm for each R-Al film, which corresponds to the absorption related to the interband transitions at the W point of the Brillouin zone in the Al band structure.^66−68^ The R-Al reflectance of the thin layer is reflective, as expected for an Al layer deposited at room temperature. However, the reflectance of the thick layer is low in comparison to the values of ∼80–90% usually reported for the Al thin layer in the visible wavelength range.^18,69−73^ In this study, the base pressure of the system was slightly higher than usual,^18,19^ which increases the impurity concentration and therefore changes the growing conditions of the Al films. This is also coherent with the R-Al surfaces reported in Figure 2 and Figure 4 and discussed previously. The B-Al films show very low diffuse reflectivity in comparison to R-Al. These values are similar to those reported in previous works.^19,21,23^ For all B-Al samples, diffuse reflectance increases with the wavelength. The B-Al absorbance increases in the solar radiation range with the increase in the film thickness to become more than 95% over the entire measuring range. The light absorption mechanism of the B-Al films was connected to two phenomena: In the first one, the nanostructure and microstructure are similar to the moth-eye antireflective structure which favors light trapping and absorbance in the bulk.^18,19^ The second one corresponds to the absorption due to the localized surface plasmon resonance (LSPR).^14,19,68,74^ As demonstrated in the previous simulations, the structure of the material is the dominant factor in visible light absorption. This implies that nanostructures with intricate morphologies and varying sizes primarily trap light through ohmic losses associated with the imaginary part of the dielectric permittivity. The LSPR band, in contrast, has a minor effect on this effect. In the case of B-Al films, the presence of nanoporosity may further slightly enhance light absorption by synergistically combining both effects.^19,25,26^

### Optical Simulation in the IR Region

**Figure 6:**
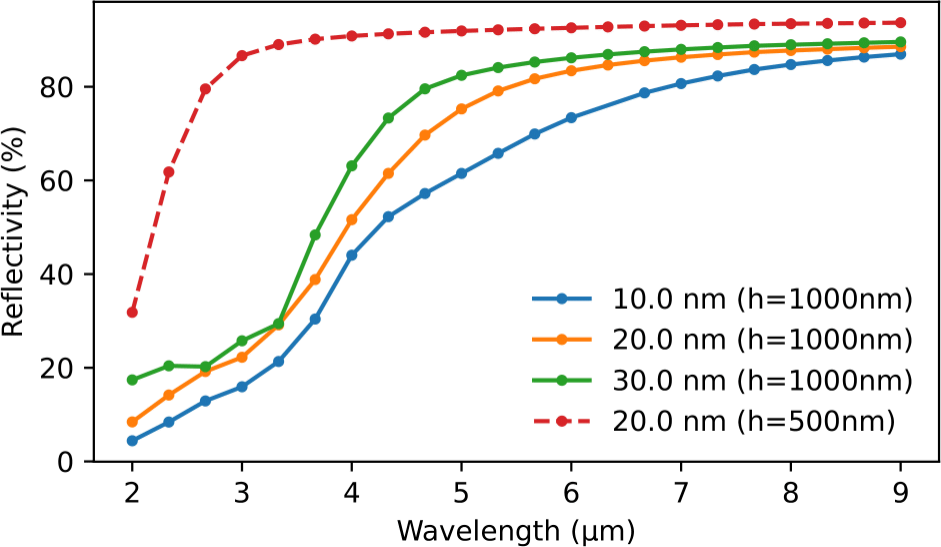
Simulated total reflectivity spectra for samples with various cell sizes for a 1-μm-thick sample. The dashed line indicates a 500-nm-thick sample.

**Figure 7:**
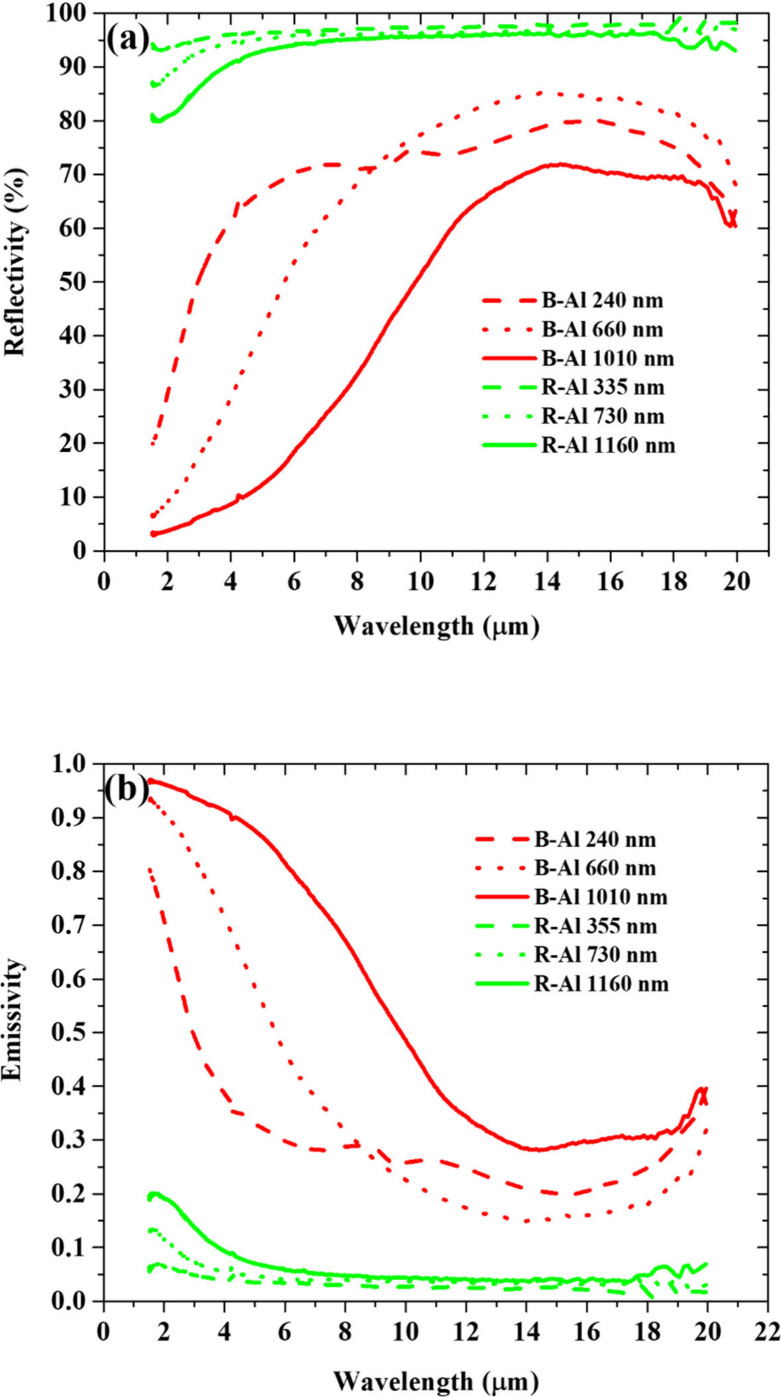
Infrared reflectivities in (a) and emissivities in (b) of R-Al and B-Al films from a 1.5 to 20 μm wavelength.

The main simulation results are shown in Figure 6, showing a transition from absorbing to reflecting behavior as the wavelength is swept from shorter to longer values. This trend matches the qualitative observations in measurements presented later in Figure 7a. To numerically study the absorption mechanism, we use finite element simulations (as detailed in the Experimental and Methodology section) to calculate the reflectivity spectrum under a normal incidence. Model structures used for the simulation are presented in the Supporting Information in Figure S2. The simulations indicate that the enhanced absorption is attributed to the porous structure, which increases the optical path length by causing light to scatter and reflect multiple times within the material, enhancing the overall absorption due to ohmic losses in the metal. In contrast, a flat aluminum surface exhibits high reflectivity, absorbing only a small fraction of the energy while reflecting most of it.

Furthermore, as illustrated in Figure 6, the reflectivity rises with increasing cell size and when the thickness of the structure is reduced. The cell size can correspond to the column dimension in the film. In the fabricated samples, these two effects counteract each other: with longer deposition times, thicker samples are obtained, but at the same time, the grain sizes increase, as depicted in Figure 3. The significant shift observed in measurements indicates that substrate thickness is the primary factor determining the cutoff wavelength for the absorptive regime of the sample.

### Optical Properties in the IR Region and Thermal Properties

Figure 7 shows the infrared reflectance (Figure 7a) as well as the emissivity (Figure 7b) of R-Al and B-Al films as a function of the wavelength from 1.5 to 20 μm. For each thickness, the R-Al layers exhibit a high reflectivity and low emissivity across the IR wavelength range, consistent with previous reports for bulk Al and Al films, where emissivity values typically range from 0.02 to 0.2.^75−78^ Variations in these values are attributed to the surface roughness of the films. In the present work, the increase in the surface roughness is related to the increase in the layer thicknesses of films measured by AFM in Figure 4. The B-Al layers present a lower reflectance and a higher emissivity than R-Al layers. The thickest B-Al layer exhibited a total emissivity of approximately 0.6. This value is lower than the typical emissivity of oxide coatings, which often exceeds 0.9.^79−82^ Black paints incorporating metal nanoparticles and metal porous coatings, typically produced through electrodeposition, also commonly exhibit total emissivities of around 0.9.^83,84^ In both cases, surface oxidation significantly enhances the measured emissivity. A key distinction between these studies and the current work lies in the thickness of the black coatings. The coatings in this study are considerably thinner than the commonly used coatings, which typically exceed several micrometers in thickness.

The reflectance and emissivity of B-Al are primarily influenced by film thickness and surface roughness, with the roughness closely related to the column dimension (cell size) that affects the infrared properties, as shown in Figure 6. For each B-Al layer, the results suggest that all layers present an average cell size below 10 nm. However, the grain size distribution extracted from the SEM images and reported in Figure 3 is much larger than the simulated one. The main reason is that these grains correspond to an aggregate of very small crystallites of ∼20 nm as observed by transmission electron microscopy and reported in our previous work.^31^ Nitrogen during deposition promotes the formation of an AlN amorphous phase at the grain boundaries. This, along with the complex nano- and microstructure, explains the lower infrared reflectivity compared to the simulation based solely on porosity. Additionally, the presence of an aluminum oxide layer on the top of B-Al layer^18^ likely enhances emissivity. Similar effects have been observed in anodized or oxidized aluminum alloys, where increased emissivity led to lower surface temperatures compared to nonanodized aluminum with the same emissivity values.^75,76^ This oxide layer on top of B-Al likely contributes to the difference between the simulated emissivity values and the experimental ones.

Table 1 reports the thermal properties of the R-Al and B-Al films as a function of their thicknesses. The R-Al films present thermal effusivities of 4554, 6255, and 6901 J·m^–2^·s^–1/2^·K^–1^ for the films of 355, 730, and 1160 nm, respectively. These values are, on average, 2.1 times higher than the B-Al thermal effusivities of 2624, 2939, and 2675 J·m^–2^·s^–1/2^·K^–1^ for the films of 240, 660, and 1010 nm thicknesses, respectively. The thermal conductivities of R-Al are 16, 35, and 35 W·m^–1^·K^–1^ for each thickness, averaging 10 times higher than the B-Al thermal conductivities of 3.0, 4.0, and 2.4 W·m^–1^·K^–1^. These values are significantly lower than the bulk Al thermal conductivity of 237 W·m^–1^·K^–1^.^85^ A common factor contributing to the lower thermal conductivities in both R-Al and B-Al films is the presence of structural defects that arise during film growth.^31,64^

**Table 1:** Thermal Properties of Each Sample Including Thermal Effusivity, Thermal Conductivity, Volumetric Heat Capacity, and Absorption Coefficient^a^

				**Absorption coefficient(m^–1^)**	
**Samples and thickness**(nm)	**Thermal effusivity(J·m^–2^·s^–1/2^·K^–1^)**	**Thermal conductivity(W·m^–1^·K^–1^)**	**Volumetric heat capacity(J·m^–3^·K^–1^)**	**IR**	**Green**
R-Al 355 nm	4554	16	1.30 × 10^6^		
R-Al 730 nm	6255	35	1.12 × 10^6^		
R-Al 1160 nm	6901	35	1.36 × 10^6^		
B-Al 240 nm	2624	3.0	2.3 × 10^6^	8.80 × 10^6^	1.76 × 10^7^
B-Al 660 nm	2939	4.0	2.16 × 10^6^	1.25 × 10^7^	2.50 × 10^7^
B-Al 1010 nm	2675	2.4	3.11 × 10^6^	9.00 × 10^6^	1.80 × 10^7^

a IR and green are wavelength areas 2 to 11 μm and 515 nm.

Polycrystalline metal films, typically deposited at room temperature, exhibit numerous grain boundaries and other defects like voids or stress in the structure of the layers.^62,64,86^ This leads to reduced electrical and thermal conductivity. This is because thermal conductivity in metals is primarily electron-mediated, and defects disrupt electron flow.^85^ The lower thermal conductivity reported in the B-Al layer can be ascribed to the higher concentration of defects and grain boundaries experimentally observed and reported in sections 2.1 and 2.2. The larger number of defects occurred during film growth due to the use of a gas mixture changing the film growth by the presence of nitrogen in the grain boundaries, which favors AlN phase formation as well as porosity.^18,19,26,31^ Although AlN has a higher thermal conductivity than Al (285 W·m^–1^·K^–1^ at 300 K for a single crystal)^87,88^ and in thin film form, AlN usually shows much lower values in the range of 0.5–30 W·m^–1^·K^–1^ due to oxygen impurities.^87^ In B-Al films, the AlN phase is predominantly amorphous, and grain boundaries contain average vacancy clusters of 5 Å.^19,26,31^ Despite these defects, B-Al films display a higher volumetric heat capacity compared to that of R-Al films, as shown in Table 1. The volumetric heat capacity usually decreases with increasing porosity due to a decrease in material density.^89^ The unexpected higher capacity can be caused by the nanostructure of the B-Al material, where for crystallites of 10 nm and smaller the specific heat increases.^90^ Radiation heat transportation that is usually nonexistent for Al is present in B-Al due to the structure and the large concentration of grain boundaries rich in AlN, which usually present a total emissivity of 0.49 at room temperature in the crystallized film.^91^ During the measurement of the thermal properties, it was observed that the B-Al films were semitransparent in both green (laser wavelength) and infrared (detector wavelengths). The evaluated absorption coefficients are in the range of 0.8 to 2.5 × 10^7^ m^–1^, which means that some amount of light is still transmitted for thicknesses larger than 100 nm. This can be caused by a connected porosity or the presence of transparent aluminum nitrides or oxides in the nanostructure.

## Conclusions

B-Al and R-Al films of varying thicknesses were deposited on fused silica substrates by using pulsed magnetron sputtering. B-Al films displayed nanocrystallized layers with smaller grain sizes and higher roughness compared to R-Al films, which had larger grain sizes and smoother surfaces. The B-Al films exhibited highly porous structures resembling cauliflower patterns, enhancing light absorption in the visible range. Simulations indicated that these porous structures contributed to high emissivity in the near- and mid-infrared, a finding confirmed by experimental measurements. The observed increase in emissivity compared with the simulated values can be attributed to the presence of AlN within the grain boundaries and the formation of an oxidized surface layer. Thermal analysis revealed that B-Al films had thermal conductivities and effusivities up to 10 times lower than those of R-Al films but higher volumetric heat capacities. Additionally, B-Al films were found to be semitransparent. These characteristics are attributed to the porous structure, nanoscale crystallites, defects, and the presence of aluminum nitrides and oxides.

## Data Availability

The main data presented in this study are available at 10.57680/asep.0602380.

## Supporting Information Available

Fit curves of the grain morphology sizes; results of XRD fits using Jana 2020 software of the XRD micrographs; and periodic unit cells for the finite element simulations (PDF)
