# Supplementary material for: Spectral Emissivity and Thermal Conductivity Properties of Black Aluminum Films
Source: Langmuir. 2025 Jan 2;41(6):3832–42. doi: 10.1021/acs.langmuir.4c03838 (PMC11841031; doi:10.1021/acs.langmuir.4c03838)
Supplement: Supplementary file 1 — la4c03838_si_001.pdf [file la4c03838_si_001.pdf]

Supporting Information

Spectral emissivity and thermal conductivity properties of Black Aluminium films.

Joris. More-Chevalier[1,2], Jiří. Martan[3], Taavi. Repän[4], Sylvain. Duprey[5], Petr. Hruška[1,6], Michal. Novotný[1,2], Petra. Honnerová[3], Jan. Kejzlar[1,2], Christophe. Labbé[5], Morgane. Poupon[1], Dejan. Prokop[1,6], Daniil. Nikitin[6], Xavier. Portier[5], Přemysl. Fitl[1,2], Julien. Cardin[5], Raivo. Jaaniso[4], Ján. Lančok[1,2].*

[1]Institute of Physics, Czech Academy of Sciences, Na Slovance 2, 182 21, Prague 8, Czech Republic

[2]Department of Physics and Measurements, University of Chemistry and Technology Prague, Technická 5, 166 28, Prague 6, Czech Republic

[3]New Technologies Research Centre (NTC), University of West Bohemia, Pilsen, Czech Republic

[4]Institute of Physics, University of Tartu, W. Ostwald St 1, Tartu, 50411, Estonia

[5]CIMAP Normandie Université, ENSICAEN, UNICAEN, CEA, UMR CNRS 6252, 6 Boulevard Maréchal Juin, 14050 Caen Cedex 4, France

[6]Charles University, Faculty of Mathematics and Physics, V Holešovičkách 2, 180 00 Prague, Czech Republic.

*Corresponding authors: morechevalier@fzu.cz

Table of contents

Figure S1: Linear fit of the mean morphology sizes…………………………………………………...S3

Table S1: Fit results of peak profiles of R-Al and B-Al layers……………………………………………S4

Figure S2: Periodic unit cells for the finite element simulations………………………………………….S5

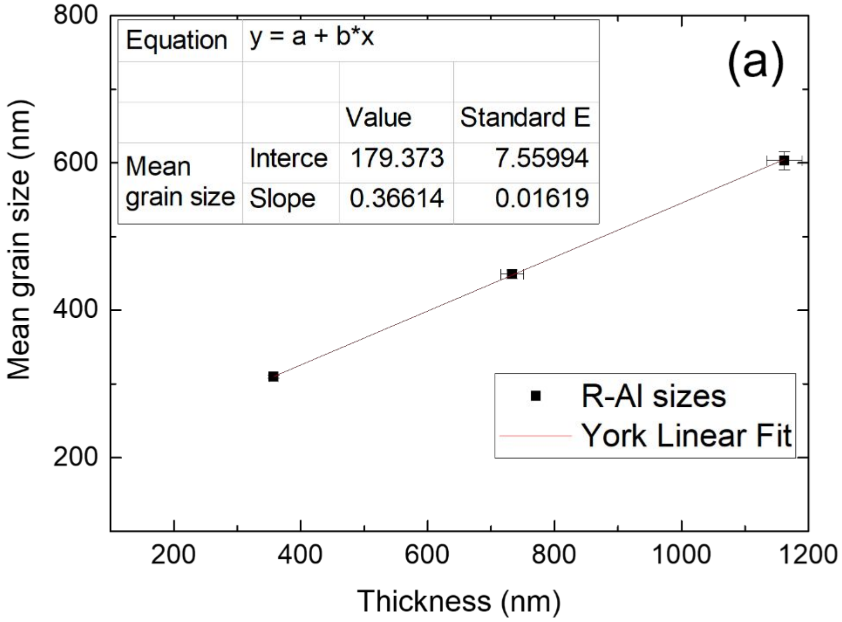

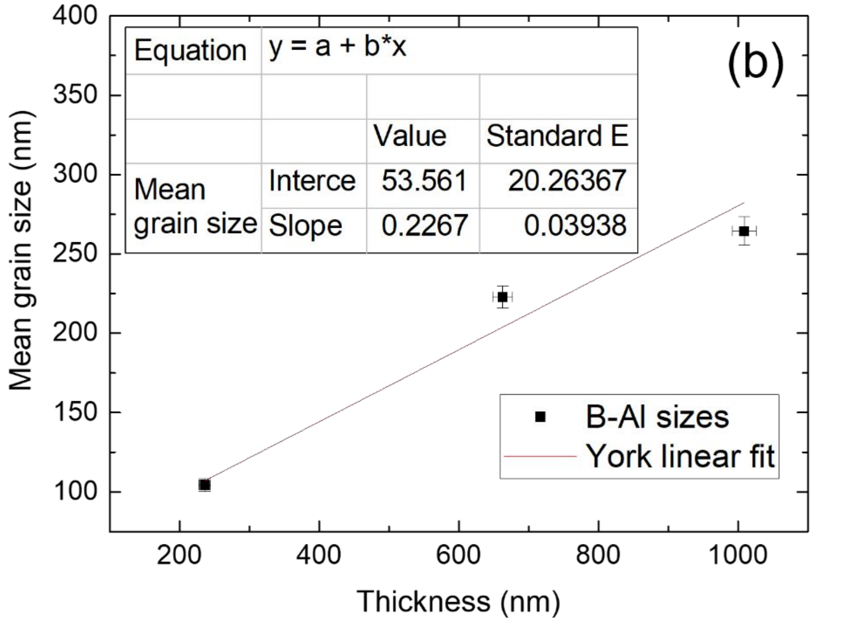

Figure S1: Linear fit of the mean morphology sizes extracted from scanning electron microscopy images. (a) Fit for R-Al film surfaces with thicknesses of 355 nm, 730 nm, and 1160 nm. (b) Fit for B-Al film surfaces with thicknesses of 240 nm, 660 nm, and 1010 nm. The error bars around the mean values represent the thickness measurement error on the x-axis and the fitting error from the log-normal distribution on the y-axis.

Table S1: Fit results of peak profiles of R-Al and B-Al layers using Jana2020 software.

R-Al 355 nm				
2θ (°)	FWHM (°)	Shift (°)<br>0.0325	a(Å)<br>4.04712	sigma<br>0.00052
38.4969	0.3149	GOF	RP	wRp
44.7497	0.3187	1.26	1.96	2.57
65.1415	0.3371	R(obs)	wR2	
78.2863	0.3553	3.81	(Obs)	
82.4976	0.3626		2.99	
R-Al 730 nm				
2θ (°)	FWHM (°)	Shift (°)<br>0.0092	a(Å)<br>4.04936	sigma<br>0.00028
38.4747	0.2923	GOF	RP	wRp
44.7235	0.2942	1.29	2.47	3.29
65.1009	0.3034	R(obs)	wR2	
78.2346	0.3126	2.96	(Obs)	
82.4419	0.3162		3.4	
R-Al 1160 nm				
2θ (°)	FWHM (°)	Shift (°)<br>-0.0147	a(Å)<br>4.04973	sigma<br>0.00019
38.471	0.2804	GOF	RP	wRp
44.7192	0.2817	1.56	2.35	3.33
65.0942	0.2882	R(obs)	wR2	
78.2261	0.2947	2.02	(Obs)	
82.4326	0.2972		2.73	
B-Al 240 nm				
2θ (°)	FWHM (°)	Shift (°)<br>0.0385	a(Å)<br>4.05144	sigma<br>0.00368
38.4378	0.7372	GOF	RP	wRp
44.6801	0.7372	1.09	1.96	2.57
65.0335	0.7372	R(obs)	wR2	
78.1488	0.7372	3.81	(Obs)	
82.3494	0.7372		2.99	
B-Al 660 nm				
2θ (°)	FWHM (°)	Shift (°)<br>0.0849	a(Å)<br>4.04830	sigma<br>0.00118
38.4852	0.3609	GOF	RP	wRp
44.7359	0.366	1.25	2.28	2.98
65.1201	0.3902	R(obs)	wR2	
78.259	0.4143	2.31	(Obs)	
82.4681	0.4239		2.38	
B-Al 1160 nm				
2θ (°)	FWHM (°)	Shift (°)<br>0.0826	a(Å)<br>4.04774	sigma<br>0.00091
38.4907	0.395	GOF	RP	wRp
44.7424	0.4026	1.22	2.51	3.26
65.1302	0.4389	R(obs)	wR2	
78.2719	0.4746	1.76	(Obs)	
82.482	0.4888		1.33	

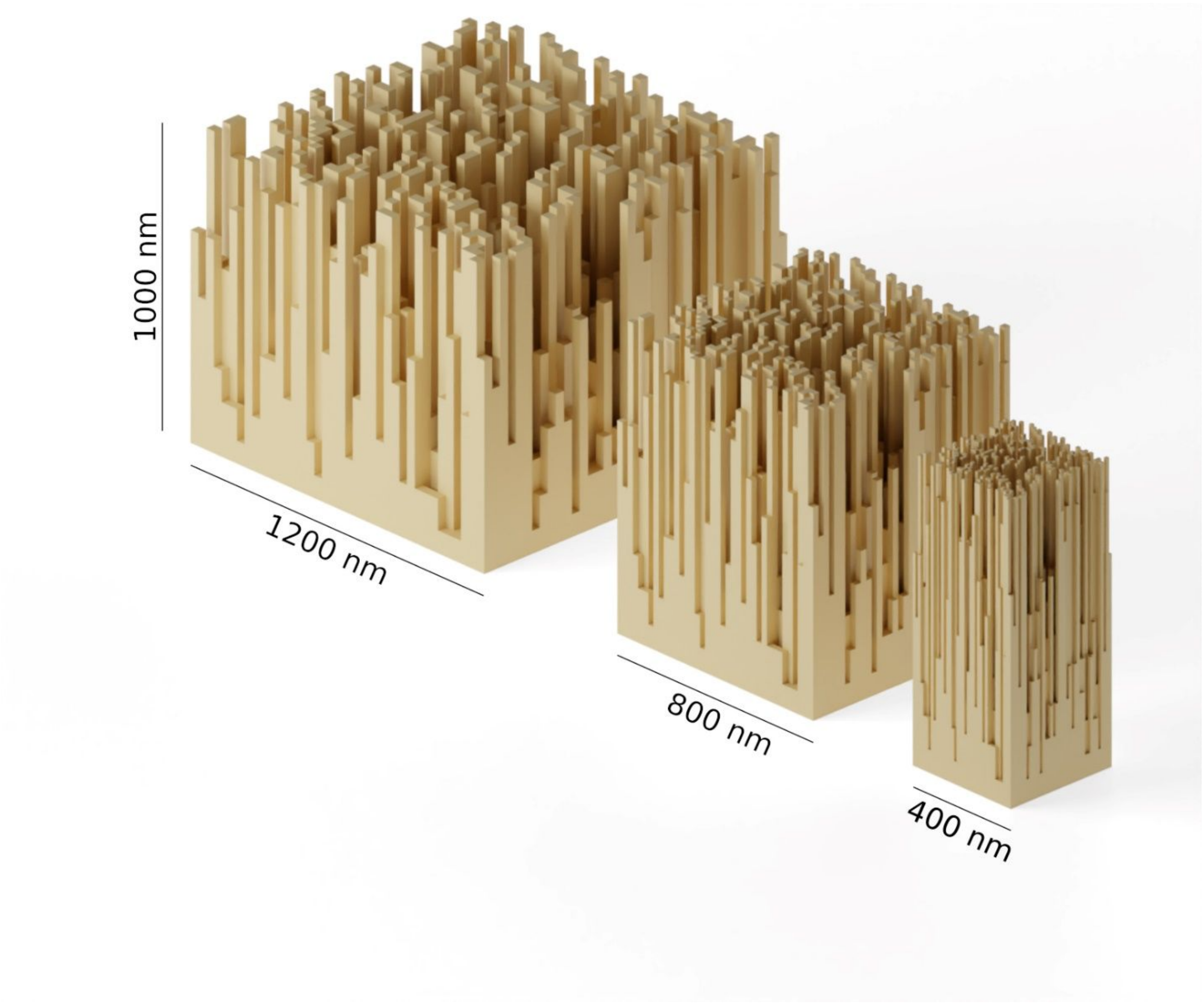

Figure S2: Periodic unit cells for the finite element simulations for h=1000 nm calculations. For the h=500 nm case, the geometry was scaled by a factor of 50% in the height axis. The geometry is based on 40×40×10 cells, so for the 400 nm × 400 nm case (on the right) the individual cells have a size of 10 nm × 10 nm × 100 nm. For these simulations, the underlying geometry is the same, apart from the scaling of the simulation domain.
